# Mydriatics in Refraction

**Published:** 1895-12

**Authors:** Richard H. Satterlee

**Affiliations:** Oculist for Buffalo, Rochester & Pittsburg Railway; Buffalo Railway Company, etc.


### MYDRIATICS IN REFRACTION.

By RICHARD H. SATTERLEE, M. D.,
Oculist for Buffalo, Rochester & Pittsburg Railway ; Buffalo Railway Company, etc.

WHILE there has been a great advance made in the various departments of medical science—and ophthalmology has been no exception to this rule—still the methods of determining refraction have hardly kept pace with this progress. It is true that we have mydriatics, which act in a much shorter time than the old two weeks' siege of atropia, but why should the oculist still cling to the routine dilatation of the pupil and the paralysed accommodation as a necessary accompaniment of the correction of refractive errors ?

The following are some of the objections to this method :

1. The dilated pupil with a paralysed accommodation is an unnatural condition of the eye. The proof of this is the fact that the patient cannot afterward wear the glass accepted under the influence of the mydriatic.

2. The cornea and lens are frequently perfect in curvature in the immediate vicinity of the normal pupil, while a short distance away they have a greater curvature in one meridian than in another. The dilated pupil would bring out these imperfections that the normal pupil would not.

3. Many people are weeks recovering from the mydriatic.

4. In some cases of beginning glaucoma the increase in tension is slight, and by no means as easily detected as the average text-book would lead one to infer. Any mydriatic in these cases produces permanent injury to the sight.

5. Hyperesthesia of the retina exists in many cases of anemia, hysteria and the like. A large pupil allows still more light to irritate the sensitive retina and greatly increases the discomfort to the patient.

6. In the majority of cases of refraction the mydriatic is unnecessary.

One or two French ophthalmologists, and a few in this country, are all that I call to mind who have discarded the routine use of mydriatics. Hypermetropia, hyperopic astigmatism, hyperopia with ciliary spasm simulating myopia—can all be determined without the use of a mydriatic. Cases of slight myopia or simple myopic astigmatism (.25 D to .75 D) require a mydriatic, but with these two exceptions it is not necessary.

Cases have been cited where dilatation of the pupil, kept up for several weeks, has relieved various reflex disturbances. A properly adjusted glass would have accomplished the same result without the danger to the retina from this increase of light.

When so much can be accomplished by glasses, should not the most advanced scientific and accurate methods be employed for the determination of refractive errors? A little more careful and conscientious work in refraction on the part of oculists would place this department much higher in the list of valuable therapeutics and send the jewelry "expert refractionist," "oculo-optician," and gentlemen of their caliber, back to mending clocks.

189 Delaware Avenue.

